# Cardiac arrest and pregnancy

**DOI:** 10.4103/0974-2700.43586

**Published:** 2009

**Authors:** Tabitha A Campbell, Tracy G Sanson

**Affiliations:** University of South Florida–Emergency Medicine, Tampa General Hospital, USA; 1 Department of Emergency Medicine, Tampa General Hospital, USA

**Keywords:** Cardiac arrest, cardiopulmonary arrest, pregnancy, resuscitation

## Abstract

Cardiopulmonary arrest in pregnancy is rare occurring in 1 in 30,000 pregnancies. When it does occur, it is important for a clinician to be familiar with the features peculiar to the pregnant state. Knowledge of the anatomic and physiologic changes of pregnancy is helpful in the treatment and diagnosis. Although the main focus should be on the mother, it should not be forgotten that there is another potential life at stake. Resuscitation of the mother is performed in the same manner as in any other patient, except for a few minor adjustments because of the changes of pregnancy. The specialties of obstetrics and neonatology should be involved early in the process to ensure appropriate treatment of both mother and the newborn. This article will explore the changes that occur in pregnancy and their impact on treatment. The common causes of maternal cardiac arrest will be discussed briefly.

## INTRODUCTION

The emergency physician may be confronted with the task of resuscitating a pregnant patient. Approximately 1 in 30,000 pregnancies are complicated by maternal cardiac arrest.[1,2] There are many causes of cardiac arrest in the general population; pregnancy increases the risks to both the mother and the fetus. Changes in maternal anatomy and physiology during pregnancy can affect both the incidence of certain diseases as well as the mother's ability to adapt to illness.[3] Trauma, pulmonary embolism, hemorrhage, hypertension, and infection are the leading causes of maternal death in pregnancy.[2,3]

## CASE REPORT

A 36-year-old female presented to the emergency department. She was gravida 3, para 1, with a twin pregnancy at 32 weeks' gestation. Her past medical history was not known. According to paramedic reports, the patient was at home when she had a change in mental status. According to family reports, she had become confused and was not acting like her typical self. When the patient became lethargic, unresponsive with labored breathing, the family called emergency medical services (EMS). Upon arrival of EMS, the patient was unresponsive, she was pulseless with asystole on the EKG monitor. She was intubated and the standard advanced cardiac life support (ACLS) protocol was followed. Enroute, she received 1 dose of vasopressin, 3 doses of epinephrine, and an amp of bicarbonate as well as 2mg of narcan without improvement in her condition. Blood glucose was 89. Chest compressions were performed throughout transportation to the hospital with no change in cardiac rhythm.. At the time of arrival at the emergency department, EMS had been performing CPR for approximately 20 min. As the patient remained pulseless, CPR was continued and an emergency perimortem cesarean section was performed. There was no change in the mother's condition following the cesarean section and she was pronounced dead. Both infants had initial Apgar scores of 2; they were intubated and resuscitated and then transferred to the neonatal intensive care unit (NICU); however, after being informed of the poor prognosis, the family decided to withdraw life support. Both infants died 2 days later. Autopsy of the mother was inconclusive, but her death was determined to be due to natural causes.

## ANATOMY AND PHYSIOLOGY OF PREGNANCY

Changes in anatomy and physiology occur throughout pregnancy and these changes affect both the presentation of certain diseases and the treatment. A sound knowledge of these changes allows the physician to determine the severity of illness and to institute the appropriate early treatment.

Pregnancy induces hemodynamic changes which can make patient assessment challenging [Table 1]. The heart rate typically increases throughout pregnancy and by the end of the third trimester the heart rate is 10–20 beats per min more than in the nonpregnant state.[1,3,5–7] Progesterone-induced smooth muscle relaxation results in decreased vascular resistance, which leads to a fall in systolic blood pressure by 2–3 mm Hg and in diastolic blood pressure by 10–15 mm Hg during the first two trimesters; the blood pressure returns to prepartum values during the third trimester.[1,4,5] There is also a 5 mm Hg decrease in central venous pressure by the third trimester.[4,6,8] Depending on the patient's prepregnancy values, all of these changes have the potential to mimic shock in an otherwise apparently stable patient. Additionally, pregnant patients have a dilutional anemia due to a 50% increase in plasma volume accompanied by a 30% increase in red blood cell mass.[1,3,4,9] At term, the placenta alone receives 10% of the maternal circulating blood volume. The increase in circulating volume means that a great amount of hemorrhage can take place before signs of maternal hypovolemia become apparent.[9]

**Table 1:** Mean values for hemodynamic changes seen throughout pregnancy

	Pre-pregnancy	1^st^ trimester	2^nd^ trimester	3^rd^ trimester
Heart rate (beats/min)	70	78	82	85
Systolic blood pressure (mm Hg)	115	112	112	115
Dlastolic blood pressure (mm Hg)	70	60	63	70
Central venous pressure (mm Hg)	9.0	7.5	4.0	3.8
Femoral venous pressure (mm Hg)	6	6	18	18
Cardiac output (L/min)	4.5	4.5	6.0	6.0
Blood volume (mL)	4000	4200	5000	5600
Uterine blood flow (mL/min)	60	600	600	600
Hematocrit (%)	40	36	34	36

At the end of the second trimester cardiac output increases by 30–50% in response to the increasing demand of the growing uterus.[1,2] There is a 10-fold increase in the blood flow to the pregnant uterus.[1,4,9] The mother's total blood volume flows through the uterus every 8–11 min.[4] Thus, placental disruption or trauma to the uterus or pelvis can result in extensive maternal hemorrhage.[9]

By 20 weeks' gestation the gravid uterus has reached the level of the inferior vena cava and in the supine position can cause vena caval compression and a resultant hypotension.[2,3,5,9,10] This can cause the supine hypotensive syndrome that is seen in approximately 10% of pregnant patients. This syndrome is characterized by syncope, hypotension, and bradycardia.[2,5,9]

The compression of pelvic veins by the enlarging uterus can cause an increase in venous pressure below the uterus. This increase in pressure explains the dependant edema, venous stasis, varicose veins, and hemorrhoids that are often seen in pregnancy. Increased venous pressure can result in rapid blood loss from injuries to the pelvis or lower extremities. Due to the increased pressure and poor venous return to the heart, intravenous lines in the lower extremities should be avoided if possible. When intravenous access below the uterus is unavoidable, it must be remembered that any medication administered through that route will have a limited return to the heart and the arterial circulation.[10]

The large increase in plasma volume during pregnancy can result in extravasation of fluid into the surrounding tissue. This is most noticeable in the lower extremities, where there is the added effect of increased venous pressure.[3] However, edema can also occur in the upper extremities, face, and oropharynx, making ventilation of the pregnant patient more difficult.[3,7,11]

Thromboembolism is more prevalent in pregnancy. Venous stasis, expanded venous volume, and increase in fibrinogen and coagulation factors are the factors that predispose to thromboembolism during the latter part of pregnancy. Immobility in a pregnant patient increases this risk and can further complicate the illness.[5,6]

In addition to the hemodynamic changes, there are also alterations in the respiratory system which can affect the patient's ability to compensate for respiratory distress. The enlarging gravid uterus slowly pushes the diaphragm more cephalad and the growing fetus puts many new demands on the maternal system.[5] There is an increase in basal metabolic rate and consequently a 15–20% increase in maternal oxygen requirements.[1] The combination of these changes cause a 40% increase in tidal volume, with a resultant 25% decrease in residual volume and functional residual capacity.[1,5,7,8,12] Therefore anoxia can occur quickly with respiratory arrest.[2] The increase in tidal volume causes increased minute ventilation and a respiratory alkalosis.[6,7] While renal compensation usually maintains a near-normal pH, arterial blood gas values may reflect an increase in pO_2_ and a decrease in both pCO_2_ and bicarbonate. Consequently, the patient is less able to buffer pH changes or to compensate for respiratory compromise, increasing the risk of injury to the fetus secondary to maternal hypoxemia and acidemia.[7]

Changes in the gastrointestinal system impact the management of the pregnant patient. The slow stretching of the abdominal wall due to uterine enlargement, desensitizes it to peritoneal irritation.[7,9] Abdominal tenderness, rebound tenderness, and guarding may be absent or significantly reduced even in the presence of significant peritoneal irritation.[9] Thus, injury or infection in the abdominal cavity may be easily overlooked. Gastrointestinal motility decreases and the gastric sphincter response is reduced, resulting in an increased likelihood of aspiration with an altered level of consciousness or during resuscitative efforts.[7,11] Moreover, increased gastric acid production during pregnancy increases the pulmonary damage following aspiration.

Changes in maternal physiology impact some laboratory values and this has to be taken into account when interpreting the results (see Table 2 for normal values in pregnancy). Lab values can be normal or falsely indicate the presence of a disease process. Hemoglobin and hematocrit will be decreased due to hemodilution. Patients may also have a slight decrease in platelet count due to hemodilution and increased consumption. White blood cells, erythrocyte sedimentation rate, and fibrinogen levels may all be increased in pregnancy.

**Table 2:** Laboratory values in pregnancy compared to controls

	Pregnancy values	Normal values
Hematocrit(%)	32-42	35-47
White blood cell count (/μ)	5,000-12,000	4,500-11,000
ESR (mm/hr)	78	<20
Arterial pH	7.40-7.45	7.35-7.44
Bicarbonate (mEq/L)	17-22	21-28
PCO_2_ (mmHg)	25-30	35-45
Fibrinogen (mg/dL)	> 400	200-400
Prothrombin time (sec)	11.2	13.5

Arterial blood gas values provide valuable information about a patient's respiratory status. A PCO_2_ of 40 is normal for the nonpregnant patient, but is a cause for concern in a pregnant patient where it may indicate poor ventilation and possible respiratory acidosis, both of which may lead to fetal distress.[5,7,13]

## PREHOSPITAL CARE

General treatment of the ill pregnant patient is largely the same as that of a nonpregnant patient; however, there are a few important differences. Any female of child-bearing age should be assessed for the possibility of pregnancy. If pregnancy is of greater than 20 weeks' gestation, the possibility of supine hypotension is increased. These patients should be placed in the left lateral decubitus position to maximize blood return to the heart.[14] The patient should be given supplemental oxygen, even in the absence of respiratory distress, to compensate for the increased oxygen demand of the fetus.[14] Intravenous access should be established for the administration of fluids and medication if necessary.

If possible, the patient should be transported to a medical center that can handle both the mother and the fetus.[9] A quick assessment of fetal viability is obtained by palpating the fundal height: when the fundus reaches the level of the umbilicus the fetus is estimated to be of 20 weeks' gestation. If the fundus is 3–4cm above the umbilicus, the fetus is potentially viable.[14,15] Appropriate arrangements, including speaking to consultants and preparation of equipment, should be made prior to arrival to the hospital in the event emergent delivery is required.

## CARDIOPULMONARY RESUSCITATION

Evaluation of airway, breathing, and circulation is the primary focus. The first priority should be resuscitation of the mother—before evaluation of the fetus is initiated. However, estimation of gestational age is useful for determining if the fetus is viable; if the fetus is deemed nonviable treatment should be directed exclusively toward maternal well-being.[9]

Fetal age can be estimated from the fundal height [Table 3]. Once the fundus reaches the level of the umbilicus, the fundal height in centimeters is approximately equivalent to the gestational age (i.e., 24 cm, 30 cm, and 34 cm measured from the symphysis pubis to the fundus is approximately equivalent to 24, 30, and 34 weeks' gestation, respectively). At 20 weeks' gestation the fundus is at the level of the umbilicus. Twenty-three to 24 weeks' gestation is considered the minimum age for fetal viability. In view of this, when the fundal height is 3–4 cm or more above the umbilicus the fetus should be considered as potentially viable.[14,15]

**Table 3:** Fundal height related to gestational age

Fundal height	Average gestational age
Pubic symphysis	12 weeks
Umbilicus	20 weeks
Xiphoid process	36 weeks

If oxygen supplementation has not been initiated prior to arrival, it should be started immediately and continued until hypoxemia, hypovolemia, and fetal distress have all been ruled out. Hypoxia can occur rapidly due to the decreased oxygen reserve and the increased oxygen consumption seen in pregnancy.[5] Maintain a low threshold for intubation. Securing the airway promotes proper oxygenation and reduces the risk of aspiration.[11,14]

The American Heart Association recommends that the standard resuscitation algorithm be followed, with a few modifications to compensate for the altered anatomy and physiology of pregnancy.[10] The primary ABC survey requires no modification to the assessment of airway or breathing for the maternal patient. Tilting the patient 15–30° to the left, or having someone pull the gravid uterus to the left, will relieve compression on the inferior vena cava and allow for optimal cardiac return.[2,5,10,16] The Cardiff wedge was designed based on the principle that an angle of 27° is optimal, allowing sufficient venous return without having a significant impact on the effectiveness of chest compressions.[2,16] No modifications are needed in positioning or dosage for defibrillation. Defibrillation does not carry any increased risk for the fetus, but the fetal monitor should be removed prior to defibrillation.[7,11]

The secondary survey requires the greatest number of modifications for the pregnant patient. Airway management in the pregnant patient can be difficult. The increased weight associated with pregnancy can result in alteration of the alignment of the larynx and difficulty in intubating. If possible, the mother should be placed in the sniffing position to facilitate intubation. In the sniffing position, the neck is flexed onto the chest and extended at the atlanto-occipital joint. The head is elevated slightly with the shoulders remaining on the table, allowing optimal alignment of the pharynx and larynx and the least amount of tongue obstruction.[18,21]

Changes in gastrointestinal motility and sphincter **tone** increase the risk of aspiration. Consider rapid sequence intubation early in the resuscitative efforts to prevent aspiration and to ensure adequate ventilation. The edematous soft tissue of the oropharynx during the latter part of pregnancy can make both mask ventilation and intubation challenging. Pregnant patients are at greater risk of developing hypoxemia, so adequate preoxygenation is essential. There is a greater rate of failed intubation in the obstetric population than in the general population.[17] As pregnancy progresses and edema of the oropharynx increases, the rate of Mallampati class III (for Mallampati classification see Table 4 below) airway increases.[1] In pregnancy the airway is more prone to injury, and trauma from attempted intubation can cause bleeding and increased swelling of the already edematous tissue.

**Table 4:** Mallampati classification of difficult airways

Mallampati classification	Visualization
Class I	Soft palate, fauces, anterior pillar and posterior pillar
Class II	Soft palate, fauces and entire uvula
Class III	Soft palate and base of uvula
Class IV	Hard palate only soft palate not visible

The special circumstances surrounding airway management in a pregnant patient should be kept in mind when intubation is considered. The induction medications and dosages are similar to that used in the nonpregnant patient. However, the mother is in a hypervolemic state and if the drugs are ineffective, higher doses may be necessary. A short-handle laryngoscope will allow easier insertion into the oropharynx. If cervical injury is not suspected the patient should be placed in the sniffing position. Cricoid pressure should be applied both to aid in intubation and to prevent aspiration. Consider using a smaller tube in view of the smaller airway and also to prevent potential complications. The use of nasotracheal intubation should be avoided due to the increased risk of injury to the edematous tissue. Likewise, an orogastric tube is preferred over a nasogastric tube for gastric decompression. Check the airway for bleeding following intubation. Alternative measures to secure the patient's airway may be necessary. Consider laryngeal mask airway if orotracheal intubation is unsuccessful; however, this does not protect against potential aspiration. Bronchoscopic intubation may be necessary, so the appropriate equipment should be readily available. Percutaneous transtracheal jet ventilation or cricothyrotomy have both proven to be lifesaving in cases where an airway could not be secured by other measures.[7,11,18]

Ventilator settings need to be adjusted to compensate for the mother's low oxygen reserve as hypoxia can occur rapidly. Both functional residual capacity and functional residual volume are decreased, and minute ventilation and tidal volume are increased, in pregnancy. Maternal oxygen saturation needs to remain above 60 mm Hg to prevent hypoxia in the fetus.[7–11]

IV access must be obtained for the administration of sufficient replacement fluids (preferably lactated Ringer's solution) and appropriate medication. Obtaining access above the uterus is essential. Due to vena caval compression, intravenous medication delivered through IV access below the uterus may not reach the heart or arterial circulation.[10] Pregnancy causes a baseline hypervolemia and the patient may require more replacement fluid than initially calculated.

The circulating maternal volume is dramatically increased, and signs of hypovolemia may not become apparent until significant hemorrhage has occurred. In a hypovolemic state the body responds with measures for self-preservation: maternal blood supply is shunted to essential organs and away from nonessential organs like the uterus; as a result, extensive fetal compromise can occur before the mother shows signs or symptoms of shock. Most ACLS medications can be given during resuscitation in pregnancy [Table 5]. However, vasopressors can impair uterine perfusion and should be avoided if possible. If after crystalloid infusion vasopressors are still warranted, they should not be withheld.[9,10,17]

**Table 5:** Resuscitative medications in pregnancy

Drug	Indications	Drug class
Epinephrine	Cardiac arrest	Category C - may induce uteroplacental vasoconstriction
Lidocaine	Ventricular ectopy, V-tach, and V-fib	Category C - may cause fetal bradycardia
Atropine	Bradycardia, asystole	Category B - can cause fetal tachycardia
Sodium bicarbonate	Cardiac arrest, metabolic acidosis	Category C
Dopamine	Hypotension	Category C - use only when clearly indicated
Dobutamine	Depressed myocardial contractility	Category C - use only if clearly indicated
Amiodarone	V-fib tachycardia, and SVT	Category D - serious fetal adverse effects have been observed
Adenosine	SVT	Class C
Magnesium sulfate	AMI, torsades de pointes, toxemia, tocolysis	Class B - neonatal neurologic depression may occur

SVT: SUPRAVENTRICULAR TACHYCARDIA, AMI: ACUTE MYOCARDIAL INFARCTION

Immediately following maternal stabilization, begin assessment of the fetus. Verification of fetal heart sounds is the first step in fetal evaluation. In the absence of fetal heart sounds upon presentation to the emergency department the chance of fetal resuscitation is poor. In this situation, focus full attention on the mother and treatment of her condition, regardless of gestational age. If fetal heart sounds are present, and the mother is stable, continue with evaluation of the fetus. The optimal fetal heart rate is between 120 and 160 beats per min.[1,3] Placental disruption or insufficiency as well as maternal hypoxia, hypovolemia, or hypotension can all result in fetal hypoxia. Hypoxia can present with fetal bradycardia. Fetal tachycardia may also be caused by fetal hypoxia, but in the presence of tachycardia fetal hypovolemia should also be considered. Bedside ultrasound is ideal for assessment of the fetus.[7,10,14,19]

## EFFECTS ON THE FETUS

The effects on the fetus of maternal cardiopulmonary arrest depend on the initiating factor. Nonetheless, maternal resuscitation offers the best hope for the fetus, no matter what the cause.[9] Fetal demise is typically due to hypoxia and can be minimized by maintaining adequate perfusion and oxygenation to the placenta.

Fetal protective measures against severe hypoxia do occur. For example, cardiac output is altered, resulting in increased blood flow to the placenta and increased gas exchange; there is also redistribution of blood volume to vital organs protecting them from severe damage. Fetal hemoglobin has a greater affinity for oxygen than maternal hemoglobin. In comparison to the maternal oxyhemoglobin dissociation curve, the fetal oxyhemoglobin dissociation curve is shifted to the left. Even at a lower partial pressure of oxygen fetal hemoglobin will bind to oxygen more strongly, allowing for greater oxygen saturation. Additionally, there is more fetal hemoglobin in each fetal red blood cell than maternal hemoglobin in maternal red blood cells, and this too allows better oxygenation of the fetus. In contrast to the mother the fetus is slightly acidotic. The acidemia results in better offloading of oxygen to the fetal tissues. As long as the maternal oxygen saturation remains above 60 mm Hg, the fetus is able to use these mechanisms to compensate for hypoxia. When the maternal oxygen saturation falls below 60 mm Hg, fetal oxygen saturation falls dramatically.[15,17]

## FETAL MONITORING

After the initial assessment of the fetus and determination of gestational age, any fetus estimated to be greater than 20 weeks' gestation (i.e., potentially viable) should be observed with fetal tocodynamometry. Initiate early fetal monitoring even if treatment of the mother is not complete. Early signs of fetal distress include tachycardia, loss of beat-to-beat or long-term variability, or late decelerations. The fetus is more sensitive to adverse conditions, and fetal distress could be an indication of impending maternal destabilization.[15]

**Figure 1:**
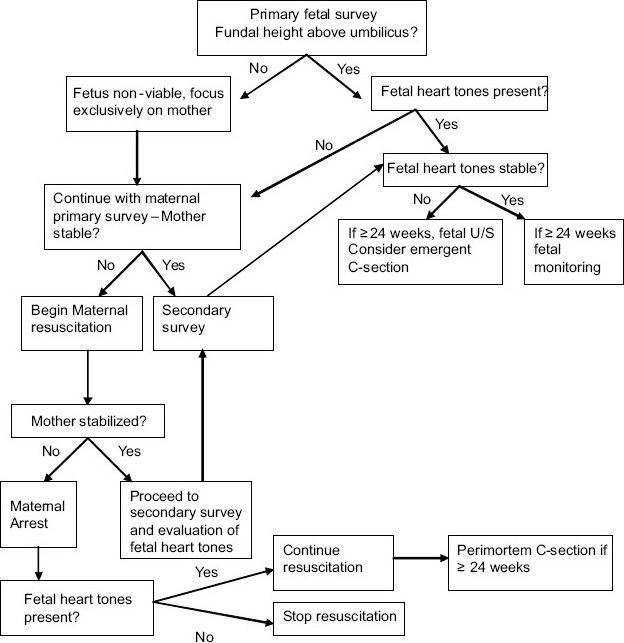
Decision making algorithm for resuscitation in pregnancy - adapted from Marx: Rosen's Emergency Medicine: Concepts and Clinical Practice, 6^th^ ed. Ch 35 Trauma in pregancy

If conditions of fetal distress persist, and the fetus is greater than 23 weeks, emergency cesarean delivery may be required. Often delivery of the fetus will result in maternal stabilization due to resolution of the cardiovascular compromise caused by aortocaval compression by the gravid uterus.[1,2,4,14] See Image 1 for a resuscitation algorithm [Figure 1].

## PERIMORTEM CESAREAN DELIVERY

If attempts at maternal resuscitation fail, consider immediate cesarean section. Gestational age greater than 23 weeks is the recommended cut-off for immediate delivery. A fetus younger than 23 weeks has an extremely poor prognosis. Such instances of perimortem delivery are rare, and the chances of fetal survival are generally poor. The earlier the fetus is delivered following maternal arrest the better is the fetal survival. Delivery should be conducted in the mother's current location; transportation to an operating room wastes valuable time. Cesarean section should be performed no later than 4 minutes after initial maternal arrest. A fetus delivered within 5 min from initiation of CPR has the best chance for survival. Deliveries occurring more than 5 min after cardiopulmonary arrest are unlikely to result in a normal viable infant.[1,4] Resuscitative efforts should continue during preparation for, and throughout, delivery of the fetus. Delivery of the fetus sometimes results in maternal recovery due to release of vena caval compression and improved cardiac return.[2,14]

## CAUSES OF MATERNAL ARREST

Although cardiopulmonary arrest in pregnancy is rare, it can occur, and identification of the underlying cause will guide further treatment. There are many causes of maternal cardiac arrest, including direct complications of pregnancy as well as preexisting disease. Table 6 lists many of the causes of cardiopulmonary arrest in pregnancy. The more common causes will be discussed in further detail.

**Table 6:** Obstetric and nonobstetric causes of cardiac arrest in pregnancy

Obstetric causes	Nonobstetric causes
Hemorrhage (17%)	Pulmonary embolism (19%)
Pregnancy induced hypertension (16%)	Infection/sepsis (13%)
Idiopathic peripartum cardiomyopathy (8%)	Stroke (5%)
Anesthetic complications (2%)	Myocardial infarction
Amniotic fluid embolism	Trauma

Trauma can complicate any pregnancy and is the most common nonobstetric cause of morbidity and mortality in pregnancy. As in all trauma patients, evaluation of airway, breathing, and circulation is the first step.

Following the primary survey and initial stabilization of the mother, the physician should proceed to the secondary survey. The secondary survey should be similar to that performed on any other trauma patient, with a few added details. Careful examination of the abdomen is essential. The enlarging uterus displaces the intra-abdominal organs making localization of injury more difficult. There is also a decreased reaction to peritoneal irritation, with reduction of tenderness, rebound tenderness, and guarding. Valuable information can still be obtained by the presence or absence of uterine contractions, uterine tenderness, or vaginal bleeding.[7,9,13,14,19]

Perform a sterile speculum examination to look for possible injuries to the genital tract, vaginal bleeding, rupture of membranes, or cervical dilation and effacement. If vaginal bleeding is obvious without speculum examination, perform an ultrasound to rule out placenta previa prior to the speculum or bimanual examination. If a large amount of vaginal fluid is present, check whether it is amniotic fluid. An alkaline pH suggests amniotic fluid, and this can be confirmed by the presence of ferning (branch-like crystals) on microscopic examination.[19]

Following the initial assessment and stabilization of the patient's condition, further tests are based on the clinical findings. As stated previously, no necessary diagnostic test should be withheld due to concern about potential adverse fetal effects. Radiography is often needed to make the appropriate diagnosis in the trauma patient. The mother is the primary patient and her survival is the best predictor of fetal survival.[9]

Ultrasound is the standard of care for the evaluation of the fetus throughout pregnancy. It is noninvasive and has no deleterious effects on the fetus. A focused abdominal sonographic examination for trauma (FAST) is often used in general trauma victims to identify intraperitoneal fluid. With minimal training, emergency physicians can assess a patient with great accuracy. In pregnancy the added benefit is that the modality allows evaluation of the uterus and fetus as well. This bedside ultrasound can be used to assess fetal movement, size, gestational age, heart rate, placental location, and amniotic fluid volume. Unfortunately the ability of ultrasound to detect placental abruption, uterine rupture, or bowel perforation is poor.[4,7,13,14,19]

With the increased availability of CT and MRI, diagnostic peritoneal lavage (DPL) has fallen out of favor with many emergency medicine physicians. It is invasive and therefore has the potential for complications. Nevertheless, when the FAST exam is equivocal, DPL has been shown to be a safe and effective alternative to CT scan in the diagnosis of intraperitoneal hemorrhage in the pregnant patient. This is especially useful during the first trimester, when radiation exposure is most deleterious to the fetus. DPL can be used to determine if a trauma patient is in need of an emergency laparotomy. If laparotomy is required, consider an open supraumbilical approach to avoid potential damage to the uterus and fetus.[7,13,14,19]

### Hemorrhage

Obstetric hemorrhage is responsible for an estimated 25% of maternal deaths in pregnancy.[17] While hemorrhage is most common during delivery and immediately postpartum, antepartum hemorrhage is also prevalent. The causes of antepartum hemorrhage include abruptio placenta, placenta previa, and uterine rupture. Although the obstetric patient is physiologically well prepared for hemorrhage due to the changes of pregnancy, there is also a greater risk of extensive blood loss from the gravid uterus. Pregnancy induces a hypervolemic state and a pregnant patient can experience massive blood loss before manifesting any significant vital sign changes. Failure to recognize and treat the hemorrhage early can result in loss of both the fetus and the mother. Rapid assessment and initiation of appropriate resuscitative measures can greatly improve prognosis.[2,9]

Placental abruption is the premature separation of the placenta from the uterine wall. There is increased risk for its occurrence with prior abruption, trauma, hypertension, cocaine abuse, smoking, premature rupture of membranes, polyhydramnios, and high parity. The elasticity of the uterus allows it to deform easily without injury; however, when the uterus changes shape, the inelastic placenta is unable to conform and is torn from the uterine wall. This abruption of the placenta causes hemorrhage between the uterine wall and the placenta. As a result fetal oxygen and nutrient supply is reduced and waste removal can be inadequate. Intrauterine hemorrhage leads to irritation of the myometrium and the uterus begins contracting. These contractions cause constriction of uterine blood vessels. resulting in a greater decrease in blood flow to the already distressed fetus. There does not appear to be a correlation between the likelihood of abruption and the location of the placenta, but the extent of abruption does correlate with the rate of fetal loss. Even a small abruption can induce premature labor; the larger the abruption, the greater is the risk to the fetus. The signs and symptoms of abruption include vaginal bleeding, uterine tenderness, abdominal cramps, maternal signs of hypovolemia, and fetal tachycardia. Although the hemorrhage is usually apparent, up to 2.51 of blood may be concealed between the myometrium and the placenta.[17] Thus, the patient can become hemodynamically unstable even with apparently minimal loss of blood.[9,15,17]

Placenta previa is the implantation of the placenta near or over the cervical os. It can also result in maternal hemorrhage, but the bleeding is rarely life threatening. Risk factors include prior previa, uterine scars, multiparity, and advanced maternal age. The bleeding occurs near the end of pregnancy as cervical effacement and dilation take place. As the lower uterine segment elongates, the placenta is separated from the uterine wall and bleeding occurs. Diagnosis is made by transabdominal or transvaginal ultrasound, which should be done prior to performing a pelvic examination. If the placenta previa is partial, the previa will resolve as the uterus stretches. As long as the mother remains hemodynamically stable the pregnancy can be carried to term. Delivery must be via cesarean section to prevent massive hemorrhage at delivery. If the mother cannot be stabilized, replacement of blood loss with fluids and immediate cesarean is indicated.[15,17]

Uterine rupture is rare but when it does occur it is associated with nearly a 100% fetal mortality rate.[4] The probability of rupture increases as the pregnancy progresses and the uterine walls are stretched. Women who have had a prior cesarean section are at highest risk of rupture from separation of the scar. The most common cause of uterine rupture is trauma. Uterine rupture should be considered when the top of the uterus cannot be palpated and/or fetal parts are easily felt through the abdominal wall. Abdominal tenderness may or may not be present. Management varies with the circumstances of the individual case. If the mother desires to have more children in the future, repair and preservation of the uterus can occasionally be accomplished. However, when there is extensive damage to the uterus or when the damaged vessels cannot be repaired, hysterectomy is the treatment of choice.[15,17]

Fetomaternal transfusion potentially occurs any time there is maternal hemorrhage. Every patient with the potential of fetomaternal hemorrhage should have blood type with Rh status determined immediately.[9,17] When fetomaternal hemorrhage is suspected in an unsensitized Rh-negative patient, the Kleihauer-Betke (KB) or the Apt tests may help in the diagnosis and management. The KB test provides a quantitative estimate of the amount of fetal blood cells present in the maternal circulation. This information is used for deciding the appropriate dose of Rh-immune globulin. In the KB test, acid elution of the sample causes the loss of hemoglobin in the maternal red blood cells causing them to appear pale (ghost cells).[15] Fetal red blood cells are stained in the process and have a bright pink appearance on microscopic examination. The fetal cells are counted, and an estimate of the fetal-to-maternal transfusion can be made. The standard dose of Rh-immune globulin is an initial 300 μg followed by an additional 300 μg for every 30 ml of estimated fetal blood transfused. This test should be repeated in 24–48 h to evaluate for continuing fetomaternal hemorrhage.[1,4,15] The KB test is very advantageous when significant fetomaternal hemorrhage occurs, but the sensitivity of the test drops substantially when there is less than 5 μl of fetal blood in the maternal circulation. The presence of as little as 0.1 μl of fetal blood can lead to isoimmunization of the mother.[1,4] The Apt test is a qualitative test used for the identification of any amount of fetal blood in the maternal circulation. When the Apt test is positive in an Rh-negative mother, a single 300-μg dose of Rh-immune globulin should be administered to prevent sensitization.[1,9,15,17]

### Embolism

Embolism causes approximately 20% of maternal deaths.[18] The majority of emboli patients are asymptomatic. Nonetheless, it is still the most common cause of acute hemodynamic and respiratory collapse in pregnancy.[18] The most common emboli are thrombi and amniotic fluid. These can enter the pulmonary circulation and result in ventilation/perfusion (V/Q) mismatch and hypoxia and consequent cardiac arrest. Rapid assessment and initiation of resuscitative measures reduces morbidity and mortality.[18]

The risk of thromboembolism increases 5- to 10-fold as a result of the venous stasis, hypercoagulability, and vascular damage (Virchow's triad) seen in pregnancy. Other contributing risk factors include: prior thromboembolism in the patient, family history of thromboembolic disease, advanced maternal age, increased parity, obesity, immobility, trauma, or recent surgery.[18] When present, the signs and symptoms of pulmonary embolism include pleuritic chest pain, cough, dyspnea, tachypnea, and tachycardia.[18]

The goal of treatment of pulmonary embolism in pregnancy is the maintenance adequate oxygenation and circulation. If pulmonary embolism is suspected, heparin should be started prior to definitive diagnosis, in addition to the usual supportive treatment. Pregnancy is a relative contraindication to thrombolytic therapy. However, it has been shown to be beneficial in some cases. It should only be used in severe cases and after careful evaluation of the risks and benefits. Diagnostic studies are ordered based on physical exam findings and clinical suspicion. Ultrasound of the lower extremities poses no risks for the fetus; unfortunately, ultrasound is not always positive in the presence of disease and other tests are often required. Deep vein thrombosis in pregnancy is more common in the iliofemoral veins and in this location is often not visible with ultrasound. V/Q scans deliver a minimal amount of radiation to the fetus and as such are safe to use in pregnancy. However, the majority of V/Q scans are intermediate risk or indeterminate, and in these cases there is still a 20% chance that the patient has a pulmonary embolism.[18] When the scan is negative, but clinical suspicion is high, pulmonary angiogram may be necessary to make the diagnosis. This procedure exposes the fetus to only minimal radiation and is safe to use in pregnancy. If venography is necessary to make a definitive diagnosis, the pelvis should be shielded to protect the fetus from radiation. Venography has the potential to induce venous thrombosis and so before its use in pregnancy the benefits should be weighed against the potential complications to both mother and fetus.[6,18]

Amniotic fluid embolus is most common immediately following delivery, though it can complicate pregnancy at any time. It is not a common complication but is associated with a high mortality rate. It is estimated that 50% of patients die within 1 h of onset and of those who survive less than half escape neurological damage.[18] Risk factors for developing amniotic fluid embolism include: difficult labor, advanced maternal age, multiparity, rupture of membranes, amnioinfusion, trauma, placental abruption, ruptured uterus, and fetal death. Unlike many other complications in pregnancy, prior amniotic fluid embolism does not appear to increase the risk for further episodes.[18]

Amniotic fluid embolism is actually an anaphylactoid reaction and not a true embolic event. When foreign debris from the amniotic fluid enters the maternal circulation a systemic inflammatory response is initiated. It typically presents with maternal respiratory distress and hypotension but can also present with shock, pulmonary edema, seizure, confusion, or coma. Patients who survive the initial insult often go on to develop disseminated intravascular coagulation (DIC) and, ultimately, multisystem organ failure. Treatment is supportive, the focus being on maintenance of oxygenation and circulation so as to minimize long-term sequelae.[6,18]

### Disseminated intravascular coagulation

DIC is a potentially life-threatening complication in pregnancy. Normal hemostasis is accomplished by a balance between coagulation factors and their inhibitors as well as thrombus formation and lysis. DIC is caused by a systemic activation of the coagulation cascade disrupting normal hemostasis. Instead of the typical local reaction to vascular damage, the body begins to uncontrollably form and lyse clots throughout the system. This results in a consumptive coagulopathy and leads to hemorrhage. Platelets are destroyed and fibrin plugs are formed which block small vessels, leading to ischemia. The ischemic changes cause further vascular damage and continuation of the coagulation cascade. Common triggers for DIC include placental abruption, fetal demise, amniotic fluid embolism, septic shock, and transfusion reactions. When diagnosing DIC, special attention should be given to the platelet value and the fibrinogen level. If these values are low, the suspicion of DIC should be high and further evaluation is needed. Treatment should focus on the underlying etiology. Most cases of DIC in pregnancy are self-limited and resolve with elimination of the trigger. If hemorrhage is extensive, patients will often benefit from replacement of platelets, clotting factors, fibrinogen, and red blood cells.[6,15]

### Eclampsia

Severe hypertension, preeclampsia, and eclampsia are a continuum of disease in pregnancy. Risk factors for pregnancy-induced hypertension include a family history of similar disease, teen pregnancy, first pregnancy, twin pregnancy, molar pregnancy, and obesity. Preeclampsia is hypertension (usually after 20 weeks' gestation) along with the presence of protein in the urine (greater than 300 mg/24 h).[20] Eclampsia is preeclampsia with added seizure activity. The cause of these various conditions in pregnancy is unknown, but is thought to be related to maternal hormone-induced vasospasm.[8] Patients present with a wide variety of signs and symptoms. Rapid weight gain, headache, visual disturbances, seizure, abdominal pain, nausea, vomiting, edema, hypertension, and hyperreflexia can all be seen in these conditions. Eclampsia should be considered in any patient with 20 weeks' gestation or greater who presents with seizures. Initial management is similar to the management of any seizure patient, i.e., secure the airway and obtain intravenous access for administration of medication. Although the mechanism of action is unknown, magnesium sulfate is the drug of choice for eclamptic seizures. Other possible causes of seizure activity, such as hypoglycemia, intoxication, etc., must be ruled out. Once the patient is stabilized, further testing is necessary to document the severity of the disease. Blood should be drawn for laboratory tests to evaluate possible end-organ damage and to look for signs of the HELLP syndrome (H – hemolysis, EL – elevated liver enzymes, and LP – low platelets). Hypertension typically resolves with the cessation of the seizure. If the diastolic blood pressure remains higher than 105 mm Hg, it may be necessary to administer an antihypertensive. The pressure should not be lowered too much as this will cause placental hypoperfusion and fetal compromise. Hydralazine is the drug of choice in pregnancy, but nimodipine and labetalol can also be used to maintain a diastolic pressure below 105 mm Hg.[21]

### Cardiac disease and acute coronary syndrome

The demands that pregnancy places on the cardiovascular system can have a dramatic impact on patients with preexisting cardiac disease. The increased cardiac output, with the increased blood volume and the relative anemia, places increased stress on the system. Previously undiagnosed disease can be revealed by this increased demand on the heart. In addition to the increased load on the cardiovascular system, pregnancy causes a hypercoagulable state. Patients are thus at increased risk of thromboembolic events as discussed in the section on pulmonary embolism. As in the nonpregnant patient, atrial fibrillation or an artificial heart valve can increase this risk further.[2,5,17,19,22]

Although rare, myocardial infarction does occur in pregnancy. As more women postpone childbearing, thus increasing the mean maternal age, the incidence of acute coronary syndrome in pregnancy has the potential to rise. Delivery should be postponed if possible following myocardial infarction. Labor and delivery put additional strain on the heart, and allowing time for the myocardium to heal improves the outcome.[2,5,22]

Treatment is similar to that in the nonpregnant patient. Standard therapy with aspirin, beta blockers, antithrombotics, and nitroglycerine are recommended. Fibrinolytics are relatively contraindicated; thus cardiac catheterization is the preferred definitive treatment.[8]

### Aortic dissection

Aortic dissection is rare in pregnancy; however, one half of all dissections in women under the age of 40 occur during pregnancy. This is thought to be due to hormonal effects on smooth muscle and connective tissue. These hormones create a laxity in the vessels and contribute to the increased risk of aortic dissection. In addition to the changes in the vessels, the elevated blood volume also contributes by causing increased pressure on the vessel walls. Aortic dissection in pregnancy should be treated in the same way as in nonpregnant patients. However, when treating aortic dissection medically, drug safety in pregnancy must be considered.[8]

## DISPOSITION

Obstetrics and neonatology should be involved early in the resuscitation. Once the patient is stabilized, care can be transferred to the appropriate specialist. If these specialties are not available, the patient should be transferred to an appropriate facility. Most patients will be admitted to the obstetric service or the intensive care unit. Continuous or intermittent fetal monitoring is necessary throughout the hospital stay.

## SUMMARY

Cardiopulmonary arrest in pregnancy can be due to nonpregnancy-related causes as well as pregnancy-related causes. Resuscitation can be challenging. With a few modifications, the basic resuscitative measures used in any patient also apply in pregnancy. A few important points to remember are as follows: anatomy and physiology are altered in pregnancy, which can change the signs and symptoms of disease and interfere with resuscitative measures. Stabilization of the mother is the primary focus and will offer the best fetal outcome. The best predictor of fetal survival when a pregnant patient suffers cardiopulmonary arrest is rapid maternal resuscitation. Prompt assessment of the maternal–fetal unit and initiation of appropriate management are essential for the survival of both patients. Fetal distress may be an early warning sign of deteriorating maternal status. No necessary test or procedure should be withheld from the mother for fear of causing damage to the fetus. Specialty services must be consulted early in the process. With a basic knowledge of the changes that occur in pregnancy, appropriate treatments can be instituted, and two lives may be saved.
